# Cholesterol Depletion from a Ceramide/Cholesterol Mixed Monolayer: A Brewster Angle Microscope Study

**DOI:** 10.1038/srep26907

**Published:** 2016-06-01

**Authors:** Pritam Mandal, Pakiza Noutsi, Sahraoui Chaieb

**Affiliations:** 1 Biological and Environmental Science and Engineering, KAUST, Thuwal, 23955, KSA; 2 Lawrence Berkeley National Laboratory, 1 cyclotron road, Mailstop 6R-2100, Berkeley, CA-94720, USA

## Abstract

Cholesterol is crucial to the mechanical properties of cell membranes that are important to cells’ behavior. Its depletion from the cell membranes could be dramatic. Among cyclodextrins (CDs), methyl beta cyclodextrin (MβCD) is the most efficient to deplete cholesterol (Chol) from biomembranes. Here, we focus on the depletion of cholesterol from a C16 ceramide/cholesterol (C16-Cer/Chol) mixed monolayer using MβCD. While the removal of cholesterol by MβCD depends on the cholesterol concentration in most mixed lipid monolayers, it does not depend very much on the concentration of cholesterol in C16-Cer/Chol monolayers. The surface pressure decay during depletion were described by a stretched exponential that suggested that the cholesterol molecules are unable to diffuse laterally and behave like static traps for the MβCD molecules. Cholesterol depletion causes morphology changes of domains but these disrupted monolayers domains seem to reform even when cholesterol level was low.

Cholesterol depletion by cyclodextrins (CDs) from biomembranes has attracted a lot of attention due to its biological and medical relevence^1^,^2^,^3^,^4^,^5^,^6^,^7^,^8^,^9^,^10^,^11^,^12^. Among the members of CD families (e.g. α-CD, β-CD, γ-CD, natural β-CD and hydroxypropyl-β-CD), MβCD is the most efficient candidate to deplete cholesterol from mixed lipid monolayers^13^,^14^,^15^. So far, a significant number of studies focused on phospholipid (e.g. DMPC/DPPC/DOPC)-mixed membranes/vesicles to test cholesterol depletion by MβCDs^1^,^4^,^16^,^17^. However, to the best of our knowledge, MβCD-mediated cholesterol depletion from a C16-ceramide/Cholesterol (C16-Cer/Chol) mixed monolayer has not been reported yet, even though C16-Cer/Chol domains are biologically relevant because C16-ceramide and cholesterol are two major components in the liquid ordered (L_0_) micro-domains (rafts) in biomembranes^18^,^19^,^20^,^21^,^22^. Cer-Chol, as well as sphingomyelin/Chol, form a scaffold in which other lipids and proteins can attach to form lipid rafts that are also liquid ordered domains^23^,^24^,^25^,^26^,^27^. These micro-domains are known to play significant roles in membrane functioning such as signal transduction or docking sites for viruses etc.^25^,^28^,^29^. It is also known that when these micro-domains grow in excess number, they can create abnormalities in membrane functioning and can cause various diseases such as Farber disease or breast cancer^23^,^30^,^31^,^32^,^33^,^34^,^35^. In particular, the number of Cer/Chol liquid-ordered (L_0_) domains increases in cell membranes in the case of Farber disease where ceramide accumulate in the cell membrane due to the lack of ceramidase^30^,^36^,^37^,^38^,^39^ Our aim is to understand the mechanism of the morphology change or the rupture of the Cer/Chol rich rafts in a model system with Cer/Chol- rich domains *in-situ*. A model system would be an easy controllable mixture in a monolayer where the temperature can be kept at ~37 °C under a lateral surface pressure of ~30 mN/m^40^,^41^.

In this work, we study the removal and depletion of cholesterol from a C16-Cer/Chol mixed monolayer using MβCD. We selected MβCD among the other members of cyclodextrin (CD) families (e.g. α-CD, β-CD, γ-CD, natural β-CD and hydroxypropyl-β-CD), because it has been reported that MβCD deplete efficiently cholesterol from mixed lipids domains^13^,^14^,^15^. We carry out the experiments at a temperature of T = 37 °C with the monolayer initially kept at a surface pressure of 30–32 mN/m because this is the lateral surface pressure of most cell membranes^40^,^41^. We spread the Cer/Chol mixture on the surface of water in a Langmuir trough that we keep at 37 °C. A monolayer forms as soon as the solvent evaporates. After we inject the MβCD into the subphase and to a final concentration of 1 mM, we monitor the monolayer behavior through a Brewster Angle Microscope (BAM) camera and its surface pressure with a Wilhemly plate. The formation of MβCD/Chol inclusion-complex as well as the consequent removal of cholesterol into the water subphase are expected to be reflected through the change in surface pressure values and domains shape changes. Any loss of material due to the depletion of cholesterol by the MβCD would result in the decay in surface pressure, and a change in domain morphology will be detected through real-time BAM imaging. Unlike earlier works where the monolayers were compressed at a rather high rate of 20 or 50 mm/min, which was detrimental to the equilibrium of the monolayer, we will compress our film at a much lower rate of 1 mm/min so that the film would be able to equilibrate and the surface pressure final value is steady^20^,^42^,^43^,^44^,^45^.

## Materials and Methods

We use cholesterol (Chol) and C16 ceramide (Cer), purchased from Avanti Lipids, to prepare the Langmuir film. To deplete cholesterol from the mixed monolayer we use commercially available methyl beta cyclodextrin (MβCD) from Sigma Aldrich. We used pure water from the Millipore-Q system (resistivity 18.2 M Ω/cm, TOC 2 ppb).

**Figure 1:**
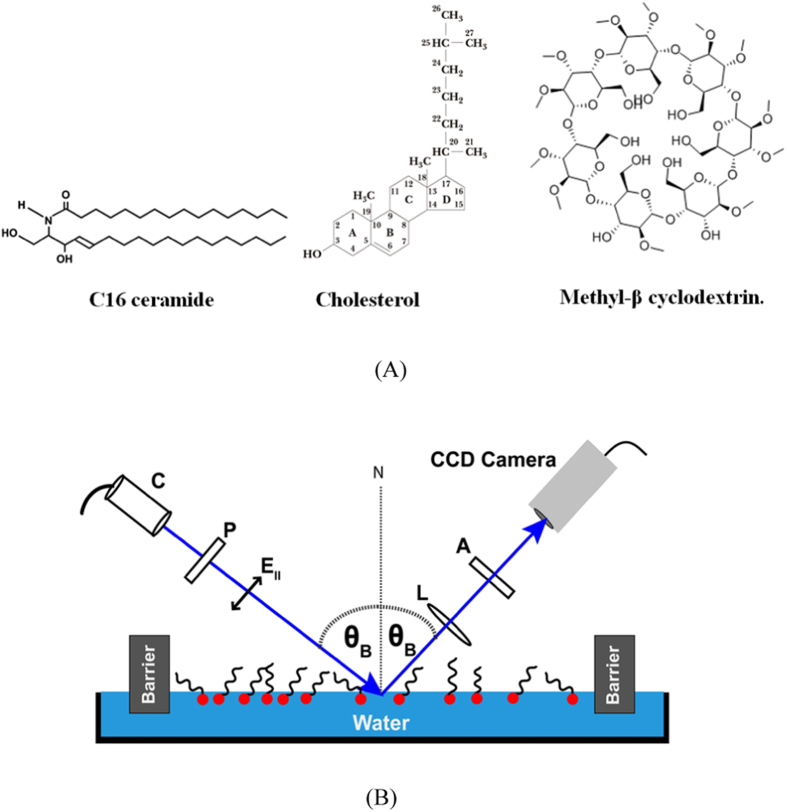
(**A**) Molecular structure of C16 Ceramide; Cholesterol and MβCD. (**B**) Schematic of BAM Experimental Set-up.

C16-Cer and Chol were dissolved in HPLC grade chloroform to prepare the stock solution and kept in the dark to prevent any light induced degradation. The water sub-phase was held in a Teflon Langmuir trough (580 × 145 × 5) mm^3^. Two symmetrically movable barriers that compress and/or decompress a monolayer were opened wide, creating an area of ~800 cm^2^ (Fig. 1B).

After the solution of mixed C16-Cer/Chol was spread on pure water which was held at T = 37 °C, we allowed ~20 minutes for the chloroform to evaporate. At low surface density of the molecules, the monolayer stays in a dilute gas phase. With symmetric compression of this monolayer from both sides, the monolayer goes from a gas phase to a compact monolayer phase. In our experiments the Chol-Ceramide Langmuir film is compressed to a compact monolayer at a speed of 1 mm/min (slow enough for the compression process to be quasi-static) up to a surface pressure of 32 mN/m. Then we leave the monolayer undisturbed for ~45 minutes to allow it to relax and the surface pressure to become stable before the MβCD solution is injected to a final concentration of 1 mM into the subphase. To prevent the disruption of the monolayer, we inject the MβCD outside the barriers. We monitor constantly the surface pressure with a stationary Wilhelmy plate balance. Any change in the surface pressure indicates a possible surface activity including loss of material from the surface. A compact Brewster Angle Microscope (BAM)^46^ from KSV NIMA, was used to directly image the air-water interface. In our compact BAM, a well-collimated *p*-polarized light with wavelength 658 nm, with 50 mW power, illuminates the surface at the Brewster angle θ_B_. At the Brewster angle the reflectivity of plane polarized (p-polarized) laser beam from an ideally plane Fresnel surface is extinct. A beam of light is said to be P-polarized light when its polarization vector lies on the plane of incidence. Any deviation from the ideal Brewster angle condition reflects the incident light from the surface and the reflectivity is no longer zero. This is the principle behind the BAM. Any addition of material that thickens the surface or any fluctuation of the interface turns the reflected light from off to on. With the introduction of a material at the interface with a different refractive index, even a molecularly thin film, a fraction of the light reflects off the film, allowing one to visualize and to image the molecularly thin film without affecting it.

## Results and Discussion

**Figure 2:**
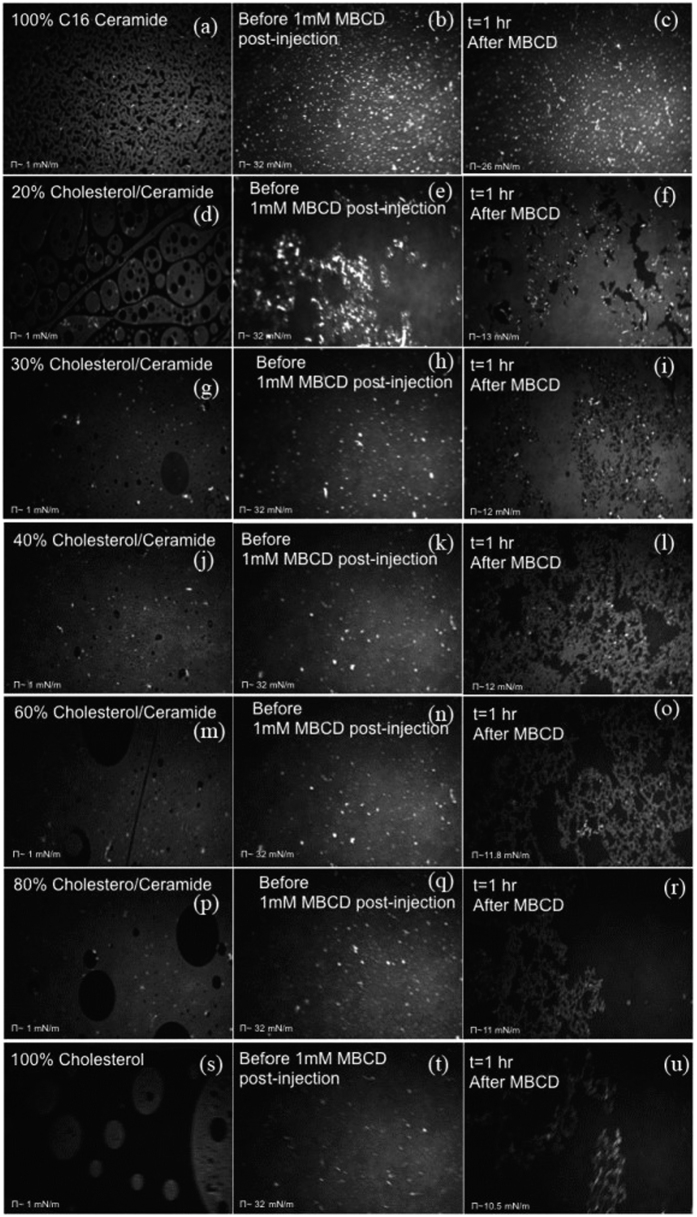
BAM images of C16 Cer/Chol mixed Langmuir film, capturing the monolayer morphology for different mole% of Chol, before and after the MβCD-injection. For each mole% of Chol, we present the monolayer at three different surface pressures: (i) very low~1 mN/m (ii) Just before MβCD-injection, ~30 mN/m, (iii) After surface pressure decay due to depletion, ~12 mN/m. In the last case, note the disrupted monolayer domains due to Chol-depletion.

**Figure 3:**
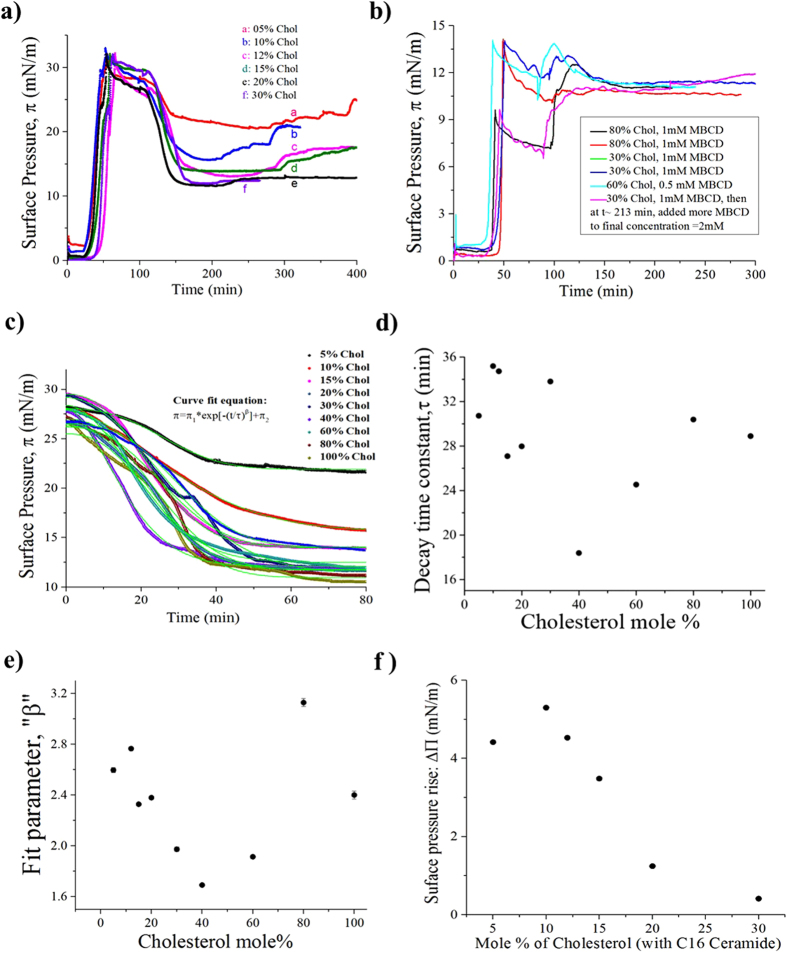
Effect of MβCD-injection in subphase: Surface pressure vs.Time for different mole% of choesterol: (**a**) The monolayer is compressed to ~32 mN/m (**b**) The monolayer is compressed to a pressure around 10 mN/m. (**c**) Fit of the surface pressure to a stretched exponential. (**d**) Decay constants (τ) vs. mole% of Chol, obtained form (**c**). (**e**) Fit parameter “β” for different mole%, obtained form (**c**). (**f**) Jump in surface pressures during domain restoration.

The concentration of cholesterol, in mol%, used in our mixed monolayer ranged from 0% to 100% in the following succession: 0%, 5%, 10%, 12%, 20%, 30%, 40%, 60%, 80% and 100%. In what follows we focus on the dependence of the cholesterol removal on its concentration in the monolayer. The efficacy of the depletion should also be reflected in the rate of decay of the surface pressure. Figure 2 shows the BAM images of domains morphology for various molar fraction of cholesterol, before and after the injection of MβCD into the subphase. For every mole fraction of cholesterol, the monolayer was spread at a very low or no surface pressure and compressed to a physiologically relevant lateral pressure (e.g. ~30–32 mN/m). After the monolayer was compressed to 32 mN/m, the MβCD was injected after about 45 minutes in order to allow the monolayer to equilibrate where the surface pressure become stable and do not fluctuate by more than 1 mN/m. Figure 2 shows also that in about 15 minutes of MβCD-injection, the surface pressure starts to decrease. As is revealed from the BAM pictures, the monolayer domains develop detectable ruptures after 30–45 minutes from the injection of MβCD. Because we constantly monitor the monolayer with BAM, we notice that the domains do not rupture simultaneously at the onset of surface-pressure decay. Figure 2 shows that by increasing the mole fraction of cholesterol, the area of disrupted regions, characterized by the presence of “holes,” increases as well. This is because the cholesterol removed increases by increasing the initial cholesterol concentration. When the concentration of cholesterol is 0% (e.g. 100% C16-Cer, Fig. 3a), the surface pressure remains nearly steady and without decreasing, suggesting that the MβCD cannot remove C16-Cer molecules into the subphase. The BAM pictures presented in the uppermost panel of Fig. 2 support this fact as well. We intuitively expected that if the number of MβCD molecules is higher than the cholesterol molecules the latter will form complexes with MβCD and dissolves into water, which leads to a decrease in the surface pressure. Instead, we found that regardless the of cholesterol concentration, the surface pressure never decayed below ~10 mN/m (Fig. 3a). This result clearly suggests that the inclusion-complex is adsorbed at the air-water interface at all times. We also conclude that a minimum amount of lateral pressure is required to squeeze out the inclusion-complex into the water subphase depleting the cholesterol from the interface. To check if the final pressure is influenced by the concentration of MβCD, we used two more concentrations of 0.5 mM and 2 mM. The results however did not change and the final surface pressure remained constant near ~10 mM/m. Further, we carry out several experiments where we first compress the monolayer to a surface pressure around 10 mN/m then we inject the MβCD beneath the monolayer to check if the surface pressure decays and if the domains become disrupted. In this instance the surface pressure first rises up to ~12.5 mN/m and then decreases to ~10 mN/m (Fig. 3b). These observations clearly show that the surface activity of the MβCD-Chol inclusion complex requires a minimum lateral pressure for the complex to be pushed out of the monolayer.

**Figure 4:**
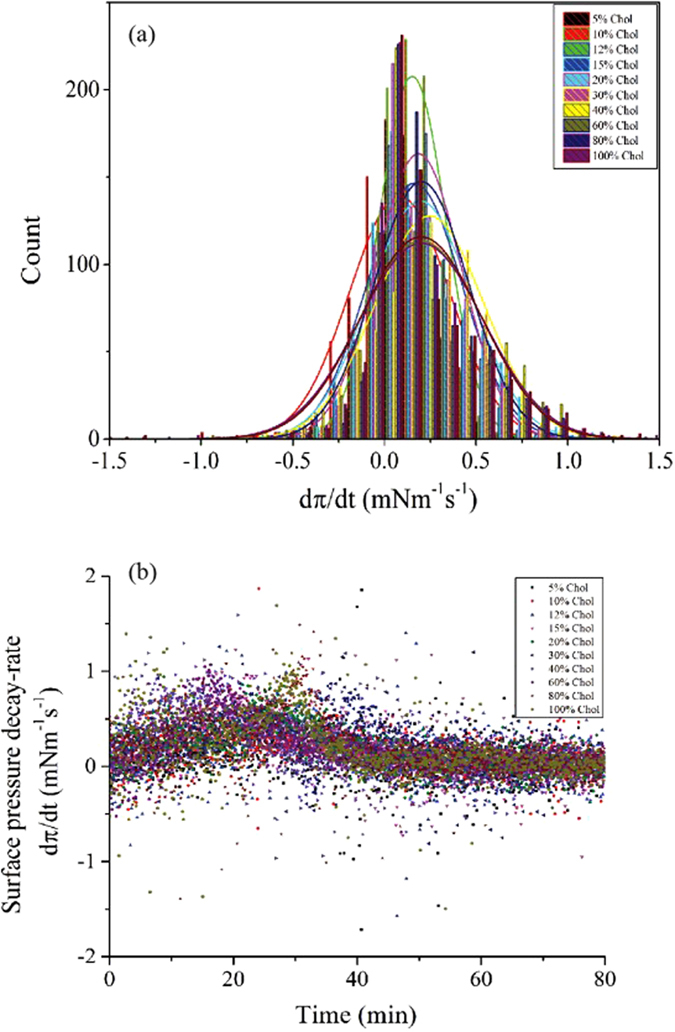
(**a**) Surface pressure decay rate (dπ/dt) as a function of time, for varying mole% of Chol. (**b**) Distribution of (dπ/dt); showing the normal Gaussian distribution centered on ~0.25 mN/m.

The relaxation of the surface pressure is best fit by a stretched exponential as shown in Fig. 3c. The expression of the stretched exponential is given in Eq. 1. The fit to an exponential is rather poor and the reason is described below. The MβCD molecules behave like random walkers and cholesterol behave like traps


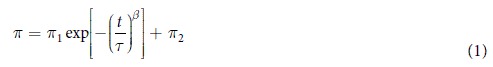


where the MβCD would “stick” drawing the cholesterol from the film into the bulk. We can think of this as a random walk with static traps^47^. It is known that if the traps, the cholesterol molecules, were dynamic and having a larger diffusion coefficient than the MβCD molecules, the surface pressure would decrease exponentially if we assume that the distribution of the cholesterol molecules is uniform and does not fluctuate too much; at least not more than the mean displacement of the MβCD molecules. This confirms that for the MβCD to deplete the cholesterol molecules from the monolayer this latter has to be compressed, which minimizes the diffusion and motion of the traps (cholesterol molecules). Figure 3b,c shows that the surface pressure’s decay during cholesterol depletion is most efficient for a cholesterol mole% around ~40%. In other studies cited above it has been mentioned that the chemical activity of cholesterol is maximum when its concentration is around 33% mole^48^. Figure 3h shows the rise (jump) in the surface pressure during domains restoration when the cholesterol mole% was below 30%. Figure 4a shows the surface pressure decay rate (dπ/dt) with time, obtained from the plot in Fig. 3c. Contrary to our expectation, (dπ/dt) vs. time for different mole% cholesterol does not drastically vary. The distribution of surface pressure decay rate (dπ/dt) shown in Fig. 4b shows the peak is around ~0.125 mMm^−1^ sec^−1^.

Now let us understand why cholesterol extraction from a C16-cer/Col mixed monolayer is best and most efficient when performed from an L_d_ or L_o_ phase. It is known that for a phospholipid/Chol mixed membrane, cholesterol is preferentially removed by MβCD from a liquid disordered (L_d_)-phase^7^,^49^. In an L_o_ phase, because of the close proximity of the phospholipids to the cholesterol molecules, the MβCD will have difficulty to reach the cholesterol molecules. On the other hand, ceramide has been found to push cholesterol out of L_o_-phase into L_d_-phase^50^,^51^, following which, one expects that an increased ceramide level would facilitate the MβCD-mediated cholesterol removal from the L_d_-phase. The depletion of cholesterol depends on the type of lipids involved^8^ (head group size, charge, dipole moment, chain length, unsaturation level etc.). MβCD binds to different lipids by absorbing their hydrophobic tails within the MβCD cavity instead of attaching to their head-groups^1^,^4^,^8^,^16^. Clearly, the attachment of cholesterol to MβCD strongly depends on the head-group size of the lipids surrounding the cholesterol molecules. Smaller head groups allow better access of the MβCD molecules to the layer, and thus ceramide molecules, which have smaller head groups, compared to phospholipids, can better attach to the MβCD’s cavity. Thus when cholesterol level is low compared to that of ceramide, the MβCD molecules can possibly attach to ceramide molecules, disrupting the condensed domains that could probably contain rafts made of Cer/Chol mixtures. At cholesterol concentrations less than 30% as compared to that of the phospholipids, cholesterol is not extracted because the larger headgroups of phospholipids shield the cholesterol molecules from being accessed by the MβCD. Whereas when the cholesterol level is higher the condensed domains are disrupted since cholesterol is depleted by MβCD. In both cases, MβCD can effectively disrupt Cer/Chol solid domains. Note that for the inclusion complex to get immersed into the subphase, the MβCD should form a dimer that is oriented perpendicular to the water-air interface where the cavity faces the surface because the length of a MβCD cavity is half the length of a cholesterol molecule^7^,^52^. When the cholesterol concentration is low, the disrupted C16-Cer/Chol monolayer domains can still be restored because both cholesterol molecules and C16-Cer molecules forms inclusion complex with the MβCD. While both Chol-MβCD as well as C16-Cer-MβCD inclusion complexes initially get drawn into the water-subphase, C16-Cer-MβCD diffuse back to the water surface restoring the holes in the monolayer that were created during depletion. This is because their hydrophobic parts that are not completely covered by MβCD, become exposed to water in the bulk. Note that the interaction of the hydrophobic parts of molecules with water increases the interaction energy, which is reduced when the hydrophobic parts are shielded out of water.

## Conclusion

The extraction of cholesterol by MβCD from a C16-Cer/Chol mixed Langmuir monolayers, held at a physiological temperature and surface pressure, revealed the following properties:

1. Unlike phospholipids/Chol system, MβCD can disrupt C16-Cer/Chol domains for all cholesterol concentrations. It can be applied on live cells at early times to detect cholesterol ceramide re-formation and thus better understand the mechanism of disease triggering.
2. As expected, the desorption-rate depends on the mole concentration of cholesterol but not drastically. For low cholesterol levels, the disrupted monolayers recover their shapes and morphology.
3. The depletion process stops when the lateral pressure of the monolayer is below 10 mN/m. The final surface pressure measured after the decay induced by the depletion, never reaches a value below 10 mN/m within experimental errors, suggesting that the MβCD inclusion-complexes accumulate at the air-water interface, forming a monolayer that requires a minimum amount of lateral pressure to be squeezed out into the water subphase.
4. When the mixed monolayer was compressed below 10 mN/m, the surface pressure first rises up to ~12.5 mN/m (after MβCD-injection), before the domains started to change shapes. This finding clearly hints that the adsorption of inclusion complex occurs at the interface. A minimum lateral surface pressure is required to push the MβCD-inclusion complexes down into the subphase.

Finally, from our study we emphasize that unlike the case of phospholipid mixed Chol membrane, in case of C16-Cer/Chol mixed monolayer domains, MβCD can efficiently disrupt solid micro domains independently of the mole% ratio of cholesterol. While we focused on the effect of cholesterol concentration on its depletion from a monolayer, a future work will be the study of the effect of the MβCD concentration on this depletion.

## Additional Information

**How to cite this article**: Mandal, P. *et al.* Cholesterol Depletion from a Ceramide/Cholesterol Mixed Monolayer: A Brewster Angle Microscope Study. *Sci. Rep.* **6**, 26907; doi: 10.1038/srep26907 (2016).
